# Supplementary material for: Periplasmic SacB as a robust counter-selection tool for genome engineering in the polyploid bacterium Zymomonas mobilis
Source: Microbiol Spectr. 2026 Jul 21;14(9):e00324-26. doi: 10.1128/spectrum.00324-26 (PMC13532227; doi:10.1128/spectrum.00324-26)
Supplement: Supplementary file 1 [file spectrum.00324-26-s0001.docx]

**Supplementary figures**

Fig. S1. Growth profiles of *Z. mobilis* strains ZM6 (wild-type) and ZM6 + *parB-sfGFP* under regular growth conditions. Biological replicates N = 3. The error bars represent the standard deviation.

Fig. S2. ParB-sfGFP localisation in the strain ZM6 + *parB-sfGFP* washed by PBS prior to imaging. The phase contrast (left panel) and fluorescence (right panel) images of the *Z. mobilis* ParB-sfGFP cells directly imaged from the culture (top) and being washed by PBS (bottom) before mounting on the agar-pad. The washed cells exhibited a dispersed sfGFP signal, indicating that washing caused dissociation of the ParB-sfGFP from the chromosomes. Scale bar, 5 μm.

Fig. S3. DNA staining of growing *Z. mobilis* cells. Growing *Z. mobilis* ZM6 cells were fixed with 4% formaldehyde and washed with PBS. The washed cells were stained with DAPI (100 μg/ml) for 15 min. The cells were spotted onto a 1% agarose-pad in PBS before imaged by fluorescence microscope. Left panel; phase contrast image. Right; fluorescence image. Scale bar, 10 μm.

Fig. S4. Time-lapse imaging of growing ZM6 + *parB-sfGFP* cells on 1% agarose-pad in the growth medium RM. Phase contrast (top panel) and fluorescence (bottom) images were taken every 10 min by fluorescence microscopy, but only selected images are shown here. Numbers in the phase contrast images indicate time (minutes) after the time-lapse imaging started. Scale bar, 5 μm

Fig. S5. ParB-sfGFP sub-cellular localisation in *Z. mobilis* cells in stationary phase. (A) Phase contrast (left) and fluorescent images (right) of ZM6 ParB-sfGFP cells that have grown overnight under standard growth conditions. Scale bars, 5 μm.

Fig. S6. ParB-sfGFP sub-cellular localisation in *Z. mobilis* grown under salt conditions. Phase contrast (left) and fluorescent (right) images of growing ZM6 ParB-sfGFP cells under salt conditions (NaCl 225 mM) conditions. Scale bar, 10 μm.

Fig. S7. Growth profiles of *Z. mobilis* strains ZM6 (wild-type) cultured in complex growth medium containing 3% sucrose as the carbon source. Biological replicates N = 2. The error bars represent the standard deviation.

Fig. S8. A plasmid map of pPK15534 + *sacB* Δ*ZZ6_0149*. The plasmid map is presented as an example to show how all plasmids were constructed in this study*.* Primers used to construct the plasmid are shown (black arrows). Primers NKF133 and 134 were used to amplify a linearised plasmid backbone. NKF135 and 136 were used to produce 500 bp upstream of targeted gene while NKF137 and 138 for the downstream. The map was obtained from the Benchling.

**Supplementary table**

Table S1. Bacterial strains and plasmids used in this study.

| Bacterial strains | |  |  | Genotype/ description/reference | | | | | |  |
| --- | --- | --- | --- | --- | --- | --- | --- | --- | --- | --- |
| *Zymomonas mobilis* ZM6 | |  |  | ATCC29191 purchased from DSMZ | | | | |  |  |
| *Escherichia coli* Dh5a | |  |  | Cloning strain (Lab stock) | | | | |  |  |
| *Escherichia coli* WM6026 | | |  | Conjugation strain (1) | | |  | |  |  |
| *Zymomonas mobilis* + SacB | | |  | *B. subtilis* *sacB* integrated at ZZ*6*_*1449 locus* (this study) | | | | | |  |
| *Zymomonas mobilis* ΔZ*Z6_1449* | | |  | Δ*ZZ6_1449* (this study) | | |  | |  |  |
| *Zymomonas mobilis ZZ6_1449-His x 6-FLAG* | | | | *ZZ6_1449-His x 6-FLAG* (this study) | | | | |  |  |
| *Zymomonas mobilis ZZ6_0812-mCherry* | | | | *ZZ6_0812-mCherry* (this study) | | | | |  |  |
| *Zymomonas mobilis ΔZZ6_1449 C-ter* | | | | *ΔZZ6_1449 C-terminus truncation* (this study) | | | | | |  |
| *Zymomonas mobilis ΔZZ6_0810* | | |  | *ΔZZ6_0810* (this study) | | |  | |  |  |
| *Zymomonas mobilis ΔZZ6_0149* | | |  | *ΔZZ6_0149* (this study) | | |  | |  |  |
| *Zymomonas mobilis ΔZZ6_1271* | | |  | *ΔZZ6_1271* (this study) | | |  | |  |  |
| *Zymomonas mobilis ΔZZ6_1369* | | |  | *ΔZZ6_1369* (this study) | | |  | |  |  |
| *Zymomonas mobilis ZZ6_1203-sfGFP (parB-sfGFP)* | | | | *sfGFP* was inserted at the C-terminus of *ZZ6_1203* (this study) | | | | |  |  |
| *Zymomonas mobilis ΔZZ6_1479* | | |  | *ΔZZ6_1479* (this study) | | |  | |  |  |
| *Zymomonas mobilis ΔZZ6_0234* | | |  | *ΔZZ6_0234* (this study) | | |  | |  |  |
|  |  |  |  |  |  | |  | |  |  |
|  |  |  |  |  | |  | |  |  |  |
| Plasmids |  |  |  | Description/reference | | | | |  |  |
| pPK15534 |  |  |  | Suicide vector (1) | | |  | |  |  |
| pPK15534 *+ sacB* | |  |  | pPK15534 carrying *B. subtilis sacB* (this study) | | | | | |  |
| pPK15534 *+ sacB ΔZZ6_1449* | | |  | pPK15534 + *sacB* carrying *Δ1449* cassettes (this study) | | | | | | |
| pPK15534 *+ sacB ZZ6_1449-His x 6-FLAG* | | | | pPK15534 + *sacB* carrying *Hisx6-FLAG* (this study) | | | | | | |
| pPK15534 *+ sacB ZZ6_0812-mCherry* | | | | pPK15534 + *sacB* carrying *0812*-*mCherry* (this study) | | | | | |  |
| pPK15534 *+ sacB ΔZZ6_1449 C-ter* | | | | pPK15534 + *sacB* carrying Δ*ZZ6_1449* C-ter (this study) | | | | | | |
| pPK15534 *+ sacB ΔZZ6_0810* | | |  | pPK15534 + *sacB* carrying Δ*ZZ6_0810* cassettes (this study) | | | | | | |
| pPK15534 *+ sacB ΔZZ6_0149* | | |  | pPK15534 + *sacB* carrying Δ*ZZ6_0149* cassettes (this study) | | | | | | |
| pPK15534 *+ sacB ΔZZ6_1271* | | |  | pPK15534 + *sacB* carrying ΔZ*Z6_1271* cassettes (this study) | | | | | | |
| pPK15534 *+ sacB ΔZZ6_1369* | | |  | pPK15534 + *sacB* carrying ΔZ*Z6_1369* cassettes (this study) | | | | | | |
| pPK15534 *+ sacB ZZ6_1203-sfGFP* | | | | pPK15534 + *sacB* carrying *ZZ6_1203-sfGFP* (this study) | | | | | | |
| pPK15534 *+ sacB ΔZZ6_1479* | | |  | pPK15534 + *sacB* carrying ΔZ*Z6_1479* cassettes (this study) | | | | | | |
| pPK15534 *+ sacB ΔZZ6_0234* | | |  | pPK15534 + *sacB* carrying ΔZ*Z6_0234* cassettes (this study) | | | | | | |
| Reference (1): Lal *et al*, Front. Microbiol. 10:2216. doi: 10.3389/fmicb.2019.02216 | | | | | | | | |  |  |

Table S2. Oligonucleotides used in this study.

| Oligo | Sequence |  |  | Use |
| --- | --- | --- | --- | --- |
| **NKF1** | TTTTTTCAACCACAAGATAACGTATGGGGCTGACTTCAGGTGC | | | **Δ*ZZ6_1449*** |
| **NKF2** | TGTTCAATGCCATCATGAATGTAATTCTCATGTTTGACAGCTTATCAC | | |  |
| **NKF3** | CCTGAAGTCAGCCCCATACGTTATCTTGTGGTTGAAAAAAATACCG | | |  |
| **NKF4** | ATTATCCCGAATTGTATTTTCAGTCTTTACTTTGTCCATCCATGACAG | | |  |
| **NKF5** | GATGGACAAAGTAAAGACTGAAAATACAATTCGGGATAATAAGGATCGG | | |  |
| **NKF6** | CTGTCAAACATGAGAATTACATTCATGATGGCATTGAACATTTCTTGGG | | |  |
| **NKF15** | GATAACGGTAAAACCGATCCCC | | |  |
| **NKF47** | AAGCCGAAGCCGATCCACCATCGTATGGGGCTGACTTCAGGTGC |  |  | ***ZZ6_1449*-*Hisx6*-*FLAG*** |
| **NKF48** | CACCTGAAGTCAGCCCCATACGATGGTGGATCGGCTTCGGCTTCGG | | |  |
| **NKF49** | TTAGTGGTGGTGGTGGTGGTGCTTGTCGTCGTCGTCCTTGTAGTCTTCCCGACCCGATGGGGTGG | | |  |
| **NKF50** | CAAGGACGACGACGACAAGCACCACCACCACCACCACTAAAAAATACAATTCGGGATAATAAGGATCGG | | |  |
| **NKF51** | GTGATGGGTTAAAAAGGATCTTGGCGAAAATGAGACGTTGATCGGC | | | ***sacB* insertion in pKK15534** |
| **NKF52** | AATTAACAGTTAACAAATAACCAGGGCTTCCCGGTATCAACAGGG | | |  |
| **NKF53** | TCAACGTCTCATTTTCGCCAAGATCCTTTTTAACCCATCACATATACCTGC | | |  |
| **NKF54** | TTGATACCGGGAAGCCCTGGTTATTTGTTAACTGTTAATTGTCCTTGTTC | | |  |
| **NKF78** | TATCTTTCATCGGGCTGATGCGTATGGGGCTGACTTCAGGTGC | | | **Δ*ZZ6_1449* C-terminus truncation** |
| **NKF79** | CCTGAAGTCAGCCCCATACGCATCAGCCCGATGAAAGATACGCC | | |  |
| **NKF80** | ATTATCCCGAATTGTATTTTCTAGATCGCCCGGTTTTGAAAAATAACGC | | |  |
| **NKF81** | TTCAAAACCGGGCGATCTAGAAAATACAATTCGGGATAATAAGGATCGG | | |  |
| **NKF91** | AAATGCCCCGCAACGATTGCCGTATGGGGCTGACTTCAGGTGC | | | **ZZ6_*0812-mCherry*** |
| **NKF92** | TGCGCTACTTCAATCGCTTTGTAATTCTCATGTTTGACAGCTTATCAC | | |  |
| **NKF93** | ACCTGAAGTCAGCCCCATACGGCAATCGTTGCGGGGCATTTAGAAC | | |  |
| **NKF94** | TCCTCCTCGCCCTTGCTCACAAACTGGTAATAAATAAAGGGCGCGAC | | |  |
| **NKF95** | GCCCTTTATTTATTACCAGTTTGTGAGCAAGGGCGAGGAGGATAAC | | |  |
| **NKF96** | CATAATGCCTTATTCCCCTACTACTTGTACAGCTCGTCCATGCCG | | |  |
| **NKF97** | TGGACGAGCTGTACAAGTAGTAGGGGAATAAGGCATTATGCCTAATGAC | | |  |
| **NKF98** | CTGTCAAACATGAGAATTACAAAGCGATTGAAGTAGCGCATGAAG | | |  |
| **NKF59** | TTTATCCGAGACGTTATCTGCGTATGGGGCTGACTTCAGGTGC | | | **Δ*ZZ6_0810*** |
| **NKF60** | AAAACGCTCACAGGCAAGAAGTAATTCTCATGTTTGACAGCTTATCAC | | |  |
| **NKF61** | CCTGAAGTCAGCCCCATACGCAGATAACGTCTCGGATAAAGAGAAG | | |  |
| **NKF62** | AATAGCCTCCCATATTAAAACGACAACTCCATAACCTGCGC | | |  |
| **NKF63** | CGCAGGTTATGGAGTTGTCGTTTTAATATGGGAGGCTATTCAGCCTC | | |  |
| **NKF64** | CTGTCAAACATGAGAATTACTTCTTGCCTGTGAGCGTTTTTGGAG | | |  |
| **NKF65** | TCTATCATGCCAATTCGCAGGC | | |  |
| **NKF133** | CAAGTGTAGAATTGTCAGGACGATCGTATGGGGCTGACTTCAGGTGC | | | **ΔZ*Z6_0149*** |
| **NKF134** | GATTAGAAGCCAATAAAGATGTAATTCTCATGTTTGACAGCTTATCAC | | |  |
| **NKF135** | GCACCTGAAGTCAGCCCCATACGATCGTCCTGACAATTCTACACTTGTCTCC | | |  |
| **NKF136** | TTTTAAGGCGCAGCTAAATCAGAGGAACTGATAAAACAGACTAAAGTTTCTTGCC | | |  |
| **NKF137** | ACTTTAGTCTGTTTTATCAGTTCCTCTGATTTAGCTGCGCCTTAAAAATAG | | |  |
| **NKF138** | AAGCTGTCAAACATGAGAATTACATCTTTATTGGCTTCTAATCCTTCTGAAAAAGG | | |  |
| **NKF150** | TTTAGGCGATGCCTATTGGACGGC | | |  |
| **NKF169** | GCTTCGCTTTTTAGCAAGGCGCCGTATGGGGCTGACTTCAGGTGC | | | **ΔZ*Z6_1271*** |
| **NKF170** | ACTTTCAGGGTCAAGAAACCGTAATTCTCATGTTTGACAGCTTATCAC | | |  |
| **NKF171** | CACCTGAAGTCAGCCCCATACGGCGCCTTGCTAAAAAGCGAAGCAG | | |  |
| **NKF172** | AGCCGTAACCGGTTTGAAAAATCAATCATGCTCACAATGTGCCTG | | |  |
| **NKF173** | GCACATTGTGAGCATGATTGATTTTTCAAACCGGTTACGGCTTTTTAC | | |  |
| **NKF174** | GCTGTCAAACATGAGAATTACGGTTTCTTGACCCTGAAAGTTTGACC | | |  |
| **NKF178** | CCTGCCATTGTCGAAGCGATGG | | |  |
| **NKF198** | AACATCGCCGCCCATCCACACCCGTATGGGGCTGACTTCAGGTGC | | | **ΔZ*Z6_1369*** |
| **NKF199** | GCGGACACTATGAACCAGAAGTAATTCTCATGTTTGACAGCTTATCAC | | |  |
| **NKF200** | CCTGAAGTCAGCCCCATACGGGTGTGGATGGGCGGCGATGTTC | | |  |
| **NKF201** | AAAACGGATTTTGAGAAGTTTAAAAGAGCCTACCTTTCTTATTACTTCAGC | | |  |
| **NKF202** | AGTAATAAGAAAGGTAGGCTCTTTTAAACTTCTCAAAATCCGTTTTAAGCGG | | |  |
| **NKF203** | AAGCTGTCAAACATGAGAATTACTTCTGGTTCATAGTGTCCGCCGC | | |  |
| **NKF257** | TGATAGGCCTTGGCTTCCTCAACGTATGGGGCTGACTTCAGGTGC | | | ***ZZ6_1203*-sf*GFP*** |
| **NKF258** | CGCTCGACAAGCTGGGTGGTGTAATTCTCATGTTTGACAGCTTATCAC | | |  |
| **NKF259** | ACCTGAAGTCAGCCCCATACGTTGAGGAAGCCAAGGCCTATCAAC | | |  |
| **NKF260** | AAAAGTTCTTCTCCTTTGCTGAAATCAGAGCCGGAAAGCCGTTGG | | |  |
| **NKF261** | GGCTTTCCGGCTCTGATTTCAGCAAAGGAGAAGAACTTTTCACTGG | | |  |
| **NKF262** | TCAATTGCGATTGGCCGGTTTTACTCGAGTTTGTAGAGCTCATCCATG | | |  |
| **NKF263** | AGCTCTACAAACTCGAGTAAAACCGGCCAATCGCAATTGATGTG | | |  |
| **NKF264** | CTGTCAAACATGAGAATTACACCACCCAGCTTGTCGAGCG | | |  |
| **NKF306** | TACCTTCGTGATTACGAAGCATCGTATGGGGCTGACTTCAGGTGC | | | **ΔZ*Z6_1479*** |
| **NKF307** | CGCGGCGCTATCGGTGATTGTAATTCTCATGTTTGACAGCTTATCAC | | |  |
| **NKF308** | ACCTGAAGTCAGCCCCATACGATGCTTCGTAATCACGAAGGTATCATG | | |  |
| **NKF309** | AAGTCGCCTTCCCTTGCCGAAGAATTCTAATTTGGCTGGTTTTGACG | | |  |
| **NKF310** | AAACCAGCCAAATTAGAATTCTTCGGCAAGGGAAGGCGACTTC | | |  |
| **NKF311** | AGCTGTCAAACATGAGAATTACAATCACCGATAGCGCCGCG | | |  |
| **NKF312** | CATTAAAGGCTTTTAACGCACGTATGGGGCTGACTTCAGGTGC | | | **Δ*ZZ6_0234*** |
| **NKF313** | CCGCTTCCTTGTTATGTCGAGTAATTCTCATGTTTGACAGCTTATCAC | | |  |
| **NKF314** | ACCTGAAGTCAGCCCCATACGTGCGTTAAAAGCCTTTAATGAGGCTG | | |  |
| **NKF315** | ACCCAGTTTACGCCATTCGGCCTTGCCCAATCCTCGTAAAAAAATGATAG | | |  |
| **NKF316** | ACGAGGATTGGGCAAGGCCGAATGGCGTAAACTGGGTCTTG | | |  |
| **NKF317** | CTGTCAAACATGAGAATTACTCGACATAACAAGGAAGCGGTAAGG | | |  |

Table S3. Primes used to detect mutation and genes.

| Deleted genes | Primers to detect mutation | | Primers to detect targeted gene | |
| --- | --- | --- | --- | --- |
| *ZZ6_1449* | NKF3 and NKF6 |  | NKF15 and NKF6 |  |
| *ZZ6_0810* | NKF61 and NKF64 |  | NKF65 and NKF64 |  |
| *ZZ6_0149* | NKF135 and NKF138 |  | NKF150 and NKF135 |  |
| *ZZ6_1271* | NKF171 and NKF174 |  | NKF178 and NKF174 |  |
| *ZZ6_1369* | NKF200 and NKF203 |  | NA |  |
| *ZZ6_1479* | NKF308 and NKF311 |  | NA |  |
| *ZZ6_0234* | NKF314 and NKF317 |  | NA |  |

Table S4. Genes deleted in this study.

| Deleted genes | Gene names | Function of gene product |  |
| --- | --- | --- | --- |
| *ZZ6_1449* | *ctpA* | carboxyl-terminal processing protease | |
| *ZZ6_0810* | *sleB* | peptidoglycan lytic transglycosylase | |
| *ZZ6_0149* | NA | Tetratricopeptide-repeat rich protein, uncharacterised | |
| *ZZ6_1271* | *popZ* | self-assembling, scaffold protein | |
| *ZZ6_1369* | NA | Acetaldehyde dehydrogenase |  |
| *ZZ6_1479* | *mltA* | peptidoglycan lytic transglycosylase | |
| *ZZ6_0234* | *mltB* | peptidoglycan lytic transglycosylase | |
